# Immunomodulatory Approaches in Diabetes-Induced Cardiorenal Syndromes

**DOI:** 10.3389/fcvm.2020.630917

**Published:** 2021-01-28

**Authors:** Lama A. Ammar, Mohamad I. Nahlawi, Nizar W. Shayya, Hilda E. Ghadieh, Nadim S. Azar, Frédéric Harb, Assaad A. Eid

**Affiliations:** ^1^Department of Anatomy, Cell Biology and Physiological Sciences, Faculty of Medicine and Medical Center, American University of Beirut, Beirut, Lebanon; ^2^American University of Beirut Diabetes, American University of Beirut, Beirut, Lebanon; ^3^Department of Life and Earth Sciences, Faculty of Sciences, Lebanese University, Fanar, Lebanon

**Keywords:** cardiorenal syndromes, diabetes mellitus, ras pathway, JAK/STAT pathway, oxidative stress, immunomodulatory approaches

## Abstract

Immunomodulatory approaches are defined as all interventions that modulate and curb the immune response of the host rather than targeting the disease itself with the aim of disease prevention or treatment. A better understanding of the immune system continues to offer innovative drug targets and methods for immunomodulatory interventions. Cardiorenal syndrome is a clinical condition that defines disorders of the heart and kidneys, both of which communicate with one another through multiple pathways in an interdependent relationship. Cardiorenal syndrome denotes the confluence of heart-kidney relationships across numerous interfaces. As such, a dysfunctional heart or kidney has the capacity to initiate disease in the other organ via common hemodynamic, neurohormonal, immunological, and/or biochemical feedback pathways. Understanding how immunomodulatory approaches are implemented in diabetes-induced cardiovascular and renal diseases is important for a promising regenerative medicine, which is the process of replacing cells, tissues or organs to establish normal function. In this article, after a brief introduction on the immunomodulatory approaches in diseases, we will be reviewing the epidemiology and classifications of cardiorenal syndrome. We will be emphasizing on the hemodynamic factors and non-hemodynamic factors linking the heart and the kidneys. In addition, we will be elaborating on the immunomodulatory pathways involved in diabetes-induced cardiorenal syndrome namely, RAS, JAK/STAT, and oxidative stress. Moreover, we will be addressing possible therapeutic approaches that target the former pathways in an attempt to modulate the immune system.

## Immunomodulatory Approaches in Diseases

Immunomodulatory approaches are defined as the all interventions that modulate and curb the immune response of the host rather than targeting the disease itself (1). With the ongoing attempts of treating infectious diseases amidst the increased pathogen resistance to traditional infectious disease control approaches, immunomodulatory interventions are being closely reviewed (1). Immunomodulatory medicines alter the response of the immune system by increasing or decreasing the production of serum antibodies, using immunostimulators and immunosuppressives, respectively (2). Immunostimulators are administered in order to enhance the immune response against infectious diseases, tumors, primary or secondary immunodeficiency, and alterations in antibody transfer. However, immunosuppressive drugs are used to reduce the immune response against transplanted organs and to treat autoimmune diseases (2). The ability of immunomodulatory approaches to modulate the immune system's disease response is evidently credited to the disruption of the proinflammatory cascade through various mechanisms involving the antioxidants effects, disruption of bacterial flora, monoclonal antibodies, cytokines, and related extracellular immune mediators and alterations in cell signaling (3). Hence, selectively either inhibiting or intensifying the specific populations and subpopulations of immune responsive cells (2).

Immunomodulatory approaches are heavily used in the medical field. In oncology, particularly for cancers unresponsive to known agents, antitumor immunotherapy is evidently decreasing fatalities through immune-cell-targeted monoclonal antibody (mAb) therapy and adoptive cellular therapy (ACT) (4). Immunomodulatory approaches have also gained attention in cardiovascular diseases by inducing inhibition to early inflammation initiators including reactive oxygen species, inhibition of mast cell degranulation and leukocyte infiltration and blocking the inflammatory cytokines and inhibiting the adaptive B and T-lymphocytes (5). That in addition to nephrology, where manipulation of the patients innate immune system, leads to the enhancement of renal repair and recovery of renal tissue hence, diminishing acute kidney injury without the progression to chronic renal diseases and consequently renal failure (6).

In this regard, understanding how immunomodulatory approaches are implemented in diabetes-induced cardiovascular and renal diseases is important for a promising regenerative medicine. Heart failure, particularly with preserved ejection fraction (HFpEF) is highly prevalent in diabetic patients (7). Several factors increase the risk of heart failure in diabetic patients. These include abnormal cardiac handling of glucose and free fatty acids (FFAs), the effect of the metabolic derangements of diabetes on the cardiovascular system and the effect of most anti-diabetic agents with glucose-lowering molecules that have direct downregulations on the cardiovascular system (8). Consequently, careful assessment should be done on how to treat patients suffering from cardiac and renal disease concurrently. This is where immunomodulatory approaches take effect given that Diabetes Mellitus (DM) is an autoimmune disease with progressive status of chronic, low-grade inflammation (LGI) (9).

Mesenchymal stem cells (MSC) are gaining large interest as being a potential approach for both heart diseases and diabetes (10). MSC are multipotent stromal cells, non-hematopoietic progenitor cells that have shown to have wide immunomodulatory capabilities by altering the adaptive and innate immunity. These capabilities are due to the natural differentiating capacity of MSCs into various different cell lineages (11). When an autoimmune disease is the target, MSC modulate the immune system of the host by inhibiting the proliferation of T cells stimulated with either polyclonal mitogens (12), allogeneic cells or specific antigens (13) through inducing an arrest to the lymphocytes at the G0/G1 phase of the cell cycle (14). For instance, Diabetic Cardiomyopathy (DCM), defined as the cardiac dysfunction that's characterized with structural, functional and metabolic changes in the myocardium that results in impaired cardiac functions (15). DCM is a distinct entity, first proposed by Lundbaek in 1954, as diabetic heart disease independent of hypertension and coronary artery disease (CAD) that are usually highly prevalent in Diabetes Mellitus (Type-2 Diabetes Mellitus) (16). In this context, chronic low-grade inflammation poses great significance in obesity and T2DM which later showed evidence in contributing to the pathogenesis of DCM (17). Mesenchymal stromal cells have been shown to have anti-diabetic as well as cardioprotective features as mentioned earlier. Studies on the mechanisms of action of mesenchymal cells showed several MSCs functions in DCM (18). One of which is the Anti-Inflammatory feature of MSCs. MSCs influence the infiltration of T cells in the pancreas along with reducing the cardiac inflammation (18). Moreover, MSCs also have the capacity to decrease cardiac tumor necrosis factor α (TNF-α) and interleukin-1 expression which both are involved in the initiation and progression of diabetic cardiomyopathy. Hence this anti-inflammatory effect of MSCs protects against myocardial inflammation under Diabetes mellitus (18).

Also, MSCs have an anti-oxidative capacity where MSCs secrete the superoxide dismutase which has the potential to treat diabetic cardiomyopathy (19). This is due to the findings showing that the overexpression of extracellular superoxide dismutase decreases macrophage infiltration and fibrosis thus leading to improved left ventricular function in the diabetic heart (18). This in addition to the anti-fibrotic features of MSCs through reducing cardiac fibrosis via weakening the survival, differentiation, proliferation, and collagen synthesis of cardiac fibroblasts (20). These features are not the only capacities of MSCs on immunomodulatory approaches to Diabetic Cardiomyopathy, others include anti-apoptotic features, pro-angiogenic and endothelial-protective features, cardiac progenitor cell-protective features and Ca2^+^ Modulating features (18).

To this end, mesenchymal stem cells “MSC” have been intensively studied as an immunomodulatory approach for the treatment of diabetes and improving the cardiovascular activity. In the context of diabetes research, Bone marrow derived MSCs have been used to form insulin-producing cells and enhance islet engraftment and survival and to treat diabetic ulcers and limb ischemia (21). In experimental models of type 2 diabetes (T2D), the mesenchymal stem cells inoculum improved metabolic control and reduced insulin requirements and of A1C with no significant opposing results after the intra-arterial injection by selective cannulation of the pancreas vasculature (21).

In a study done by Si et al., rat models with induced T2D were used to investigate the effects of autologous MSC inoculum. The autologous MSCs were injected shortly “1 or 3 weeks” after the streptozotocin treatment. streptozotocin “STZ” is used to induce a hyperglycaemic state to induce diabetes' (22). The results showed improved metabolic control through enriched insulin secretion, amelioration of insulin insensitivity and increased islet numbers in the pancreas. These results are in consistency with research evidence on the potential therapeutic properties of MSCs to treat diabetes (22). The potential capacity of bone marrow derived MSCs in enhancing the cardiovascular activity in diabetic cardiomyopathy was also investigated and has been shown to lie in its direct differentiation to cardiomyocytes and the ability to secrete potent trophic and paracrine mediators which induces cardiac regeneration and cardio protection (23). Studies on rats with type 1 DM, intravenous administration of bone-marrow derived MSCs has shown to improve cardiac function by increasing angiogenesis and attenuating cardiac remodeling. This is attributed to the differentiation of the transplanted MSCs into cardiomyocytes and improved angiogenesis and myogenesis therefore increasing matrix metalloproteinases MMP-2 activity, decreasing that of MMP9 and reducing collagen load in the diabetic myocardium (24).

After this overview of various immunomodulatory approaches related to Cardiovascular Diseases and especially Diabetic Cardiomyopathy, we will look at the immunomodulatory approaches to a Renal Disease: Diabetic Nephropathy (DN). The latter is a syndrome characterized by the presence of pathological quantities of urine albumin excretion, diabetic glomerular lesions, and loss of glomerular filtration rate (GFR), thickening of the glomerular basement membrane (GBM) in diabetic patients (25). Many immunomodulatory approaches are proposed to deal with DN. One of which is also MSCs. In a study done at the Kunming Medical University showed that MSCs ameliorate the renal function and extend survival in diabetic rats, regulate the production of lipoxin 4 (LXA4) and ALX/FPR2 (the receptor of LXA4) in kidney tissue of DN, protect renal function and inhibit fibrosis (26). Other approaches include the novel bifunctional cytokine Interleukin-233 (IL-233) that bears IL-2 and IL-33 activities that reverses inflammation and protects against Type 2 Diabetic Nephropathy by promoting T-regulatory cells (Treg) and type 2 immune response. IL-233 also attenuates hyperglycaemia and proteinuria, preserves renal structure and function for long-term and restores glucose clearance and inhibits visceral adiposity (27).

The high prevalence of this syndrome entails the discovery of new treatments to curb the progression of the disease. Because of a better understanding of the disease, there have been many advances in immunomodulatory approaches used for treating diabetes-based cardiorenal diseases. As such, currently there are improvements in the applications of this knowledge to clinical settings, which have led to treatments that are more effective. Since treatments with single agents did not achieve stable metabolic remission as such, future immunomodulatory approaches would focus on dosing, timing, and recognition of the differences between different species. As a result, the next step would be to focus on combined therapy to improve the efficacy of the treatment by promoting additive effects.

In this regard, in what follows we tackle the cardiorenal syndrome; epidemiology & classification, the relation between the cardiac and renal systems and the pathways involved in cardiorenal syndrome in diabetes namely, RAS, JAK/STAT and oxidative stress.

## Cardiorenal Syndrome

The homeostasis in the human body is maintained by the coordinated work of several organs and systems. The most important key players in the homeostasis are the heart and the kidneys. The heart is the pump of the body, which is responsible for circulating blood within the body. On the other hand, the kidneys are responsible for filtering the blood and for the electrolyte homeostasis. These two organs are interlinked; where a dysfunction in one organ affects the other. From this tight relation emerges the cardiorenal syndrome. According to Ronco et al. (28) the cardiorenal syndrome is “the disorders of the heart and kidneys where one organ affects the other.” Another more holistic definition was stated by Bock and Gottlieb (29) in their article in which they have mentioned that the cardiorenal syndrome is when “each dysfunctional organ (heart and kidneys) has the ability to initiate and perpetuate disease in the other organ through common hemodynamic, neurohormonal, immunological, and/or biochemical feedback pathways.” Although these two definitions have been established around 10 years ago, to this day a clear mechanistic understanding of the cardiorenal syndrome is not yet agreed upon (30).

### Epidemiology of Cardiorenal Syndrome

It is estimated that 25 to 60% of patients with heart failure have some type of cardiorenal syndrome which is associated with high morbidity and mortality (31). In the United States, the prevalence of chronic heart disease is estimated at 2% of people over 45 of age. In comparison, in end stage renal disease, 30% of the patients have chronic heart disease upon the initiation of dialysis (32) and cardiovascular disease is the most common cause of death in those patients (33).

### Classifications of Cardiorenal Syndrome

In 2008, a consensus conference on cardio-renal syndromes was held in Venice Italy under the umbrella of the Acute Dialysis Quality Initiative (ADQI) (28). In this conference the classification of the types of the cardiorenal disease were agreed upon. These classifications are considered clinical which lack structural and functional analysis of the disease mechanisms and therapeutic options (34). However, these classifications are useful as a first step to reach a functional classification (35). The classification system is divided into five types of cardiorenal syndrome (28).

#### Acute Cardio-Renal Syndrome (Type 1)

This type is characterized by the acute worsening of heart function leading to kidney injury and/or dysfunction. Acute worsening of heart function might include pulmonary oedema, cardiogenic shock, acute heart failure (HF). Approximately 27 to 40% of patients admitted with acute heart failure develop acute kidney failure (36).

#### Chronic Cardio-Renal Syndrome (Type 2)

This type is similar to the first type, except that the heart failure or dysfunction here is chronic which leads to kidney failure. This is the most common type of cardiorenal syndrome. It has been reported that 63% of patients admitted with congestive heart failure have type 2 cardiorenal syndrome (37).

#### Acute Cardio-Renal Syndrome (Type 3)

This type is the opposite of type one, wherein the acute renal impairment causes acute cardiac dysfunction or failure. The epidemiology of this subtype has proven to be a challenge to define due to the wide definition of acute kidney injury and limited reporting on it (28).

#### Chronic Cardio-Renal Syndrome (Type 4)

This type is characterized by chronic kidney injury leading to cardiac injury, disease, and/or dysfunction, such as left ventricular failure or diastolic heart failure. There is a strong correlation between the severity of the kidney injury and the unfavorable heart outcomes (38).

#### Secondary Cardiorenal Syndrome (Type 5)

This type is characterized by the simultaneous cardiac and renal dysfunction that is caused by a systemic condition that may be chronic or acute. These systemic conditions include sepsis, diabetes mellitus, amyloidosis, and other chronic inflammatory conditions (28).

## Cardiorenal Syndrome in Diabetes

**Figure 1:**
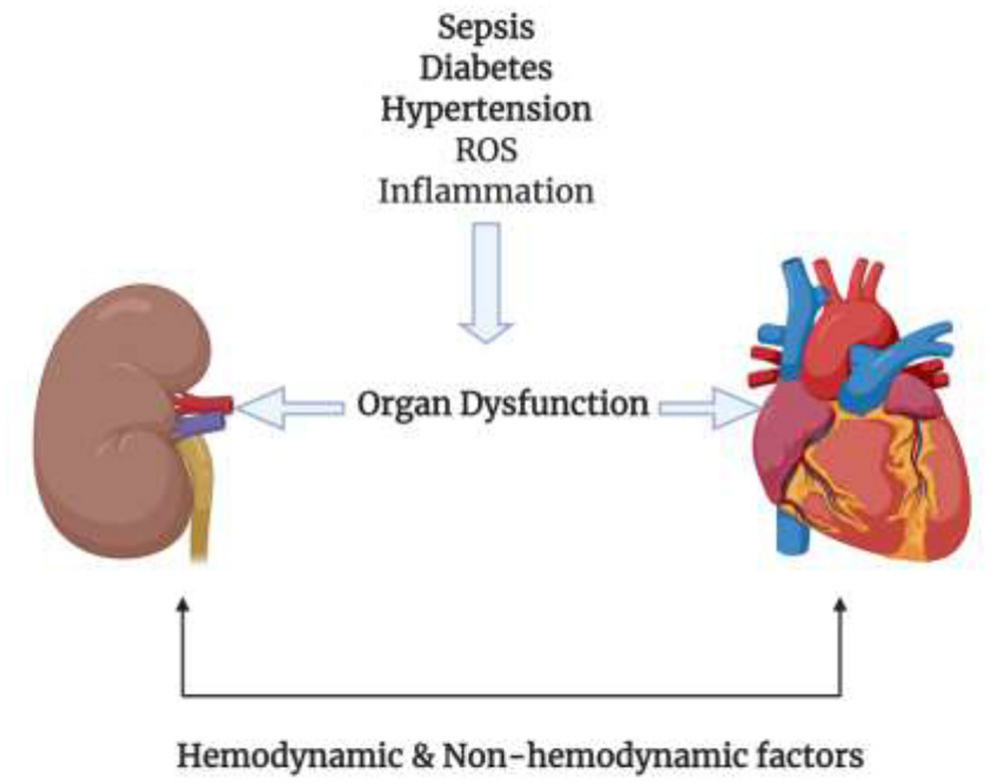
Different conditions such as Sepsis, diabetes, hypertension, ROS, and inflammation cause damage to the kidney or heart where one organ has the ability to injure the other organ by the means of hemodynamic and non-hemodynamic factors linking the heart and the kidneys.

The relation between the heart and the kidneys is a pretty complex one. Many factors, including pathways, molecules, and dysfunctions, come at play to make this tight link. It is hard to study each factor on its own because of their interlinked nature; however, some articles were able to classify these factors into hemodynamic factors and non-hemodynamic factors, as shown in Figure 1 (34). It is important to keep in mind that although this classification is in place, these factors are not independent of each other. It is also evident that these two factors have a strong relation with diabetes. Most of the diabetic complications are due to the macro and microvascular injury, that in turn affect the hemodynamic factors (39). On the other hand, in diabetes the production of mitochondrial ROS plays a huge role in the pathogenesis of diabetes and the development of its complications (40). This lies under the umbrella of non-hemodynamic factors. Moreover, fibrosis is a unifying mechanism linking cardiorenal syndromes. Fibrosis is a result of many metabolic derangements, whether in the heart or in the kidney, which eventually lead to cardiorenal syndromes.

### Hemodynamic Factors and Non-hemodynamic Factors

#### Hemodynamic Factors

Hemodynamics, or in other words dynamics of the blood flow, are tightly controlled by homeostatic mechanisms. Dysfunctions in this well-maintained system have adverse effects on numerous organs and tissues especially the kidneys. The renal blood flow is the primary driver of the glomerular filtration rate (GFR). According to the well-known equation, the GFR equals the renal plasma flow times the filtration fraction. Moreover, many articles that date as back as the mid twentieth century have established that reduced renal blood flow and increased central venous pressure are primary effector mechanisms for renal impairment (38, 41). Later studies suggested the renal autoregulation phenomenon, where the reduction in renal blood flow (RBF) was out of proportion to the reduction in cardiac index, while GFR was relatively maintained (42). However, when the renal blood flow drops further, GFR declines as autoregulatory capacity is exhausted (43). In the last few years, the research focus has shifted to the venous congestion as another important factor in the drop of theGFR, independent of the renal blood flow (44).

#### Non-hemodynamic Factors

As stated before, hemodynamic and non-hemodynamic factors are not independent of each other. It is very hard to study the effect of one factor in isolation of the other. Especially that the non-hemodynamic factors, also called cardiorenal connectors, act on the glomerular filtration rate by changes on the hemodynamics. Thus, these cardiorenal connectors are more mediators than direct effectors. The non-hemodynamic factors include a wide range of factors that include the renin angiotensin system (RAS), sympathetic nervous system (SNS) activation, inflammation, endothelial dysfunction.

The RAS is considered a prototypical cardiorenal connector since it is activated bidirectionally by the heart and the kidneys upon failure. Renin is released when renal artery pressure is decreased (45), renal venous pressure is increased (46) and when the delivery of sodium to the distal nephron is decreased which all occur in heart failure and chronic kidney disease. Moreover, angiotensin II has an important effect on renal perfusion and it promotes renal fibrosis, which directly affects GFR, induces hypo-responsiveness to natriuretic peptide and mediates SNS activation (47). The SNS is responsible for altering the ultrafiltration coefficient and is associated with tubular injury and the formation of reactive oxygen species (ROS) (40). Angiotensin II is also responsible for modulating oxidative stress and endothelial dysfunction. Through Nicotinamide Adenine Dinucleotide Phosphate [NADP(H)] activation, angiotensin II promotes the formation of reactive oxygen species, which can cause intrarenal (proximal tubular) damage (33).

### Fibrosis as a Unifying Pathophysiology of the Cardiorenal Syndromes

Fibrosis is a complex cascade of cellular and molecular processes caused by disease related injury. Over a short period of time, fibrosis serves as an adaptive process that helps the organ. However, over an extended period of time, fibrosis will cause parenchymal scarring and ultimately cellular dysfunction and organ failure (48). The main cause of fibrosis in the heart and kidney is inflammation- and oxidative stress–related endothelial dysfunction in aging, hypertension, diabetes mellitus, and obesity (49).

As much as the details described above shows how the heart and kidney are affected, it is of interest to determine the possible pathways that could be implicated in the cardiorenal syndromes.

## Immunomodulatory Pathways Involved in Cardiorenal Syndrome in Diabetes

### RAS Pathway

**Figure 2:**
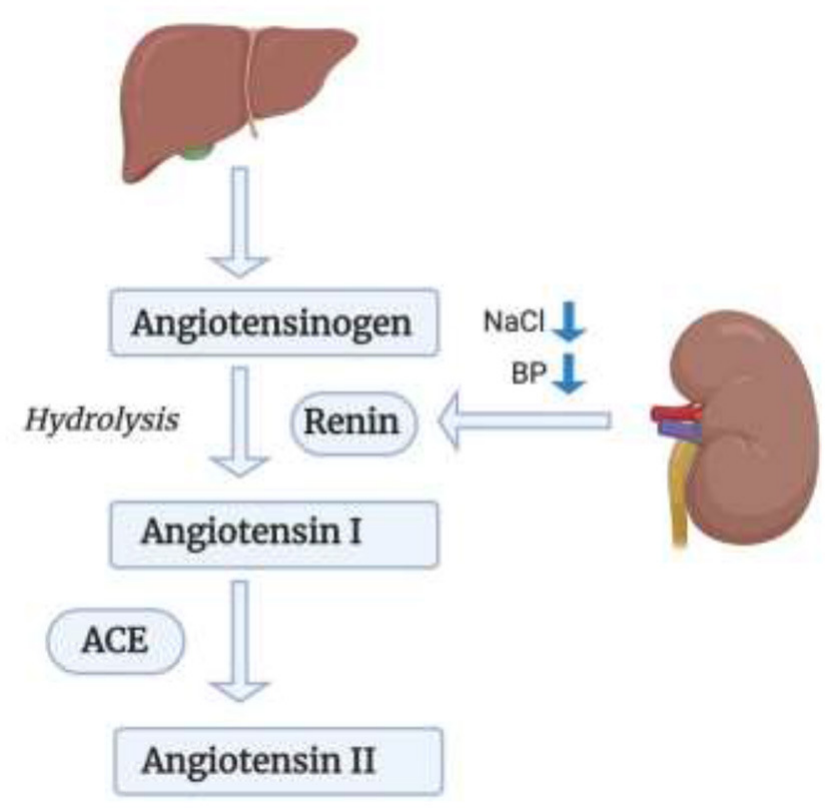
RAS pathway in leading kidney failure. Renin acts on Angiotensinogen secreted by the liver, transforming it to Angiotensin I, which will then be transformed into Angiotensin II via ACE.

RAS is one of the most important cardiorenal connectors. Improper activation of RAS can lead to both heart and renal failure. In heart failure, RAS alongside the SNS are overactivated (50). Our focus in this section will be Angiotensin II which is the most important effector molecule in the RAS pathway, shown in Figure 2 (51). In response to a drop in blood pressure and/or sodium chloride (NaCl) level, Renin which is also known as Angiotensinogenase, is secreted by the Juxtaglomerular apparatus in the kidneys (52). It is also secreted in response to SNS activity via the β-1-adrenoceptor activation by norepinephrine secretion which induces inflammation via LPS-induced IL-6 production (52–54). Renin acts on Angiotensinogen that is secreted by the liver transforming it to Angiotensin I via hydrolysis; which will then be transformed to Angiotensin II by the action of Angiotensin Converting Enzyme (ACE) that will have multiple effector sites (52). Angiotensin II, upon binding to its AT-1 (Angiotensin II type 1 receptor) and AT-2 receptor (Angiotensin II type 2 receptor) (53, 55), induces the production of Interleukin-6 (IL-6) and of the TNF-α via the Protein Kinase C (PKC) pathway, followed by the activation of two transcription factors: first of which is the Nuclear Factor Kappa B (NF-κB) through phosphorylation of p65 and then the Activator Protein 1 (AP-1) (53). These two transcription factors are important in the pathway for expression of TNF-α and thus for inducing an inflammatory response. This might be an important future perspective in the crosstalk between inflammatory cytokines and RAS in the heart. Another important cytokine induced by Angiotensin II binding to the AT-1 receptor is IL-1β. IL-1β plays a role in heart failure via systolic dysfunction and ventricular remodeling by upregulating Transforming Growth Factor-Beta (TGF-β) (56). Il-1β impairs systolic function by decreasing the expression of genes important in the regulation of calcium homeostasis (57). It also increases Nitric Oxide Synthase (NOS) expression in cardiac myocytes which leads to an increase in Nitric Oxide (NO) activity, a decrease in energy production and a lower myocardial contractility (57). In addition to that, according to a study performed on diabetic mice, IL-1β leads to cardiac arrhythmia by causing a prolongation of action potential duration, a decrease in potassium current and an increase in Calcium sparks in cardiomyocytes (58). IL-1β also affects the synchronized contraction of the heart by decreasing the expression of Connexin 43 (Cx43), a major protein in the cardiac gap junctions (57). Additionally, Angiotensin II induces the production of yet another cytokine, IL-17 via AT-1 receptor binding (59). IL-17, which is produced by T-helper 17 (Th17) cells, triggers the production of other proinflammatory cytokines such as IL-6 and TNF-α, and contributes to the pathogenesis of hypertension and atherosclerosis; as well as, insulin resistance (59).

**Figure 3:**
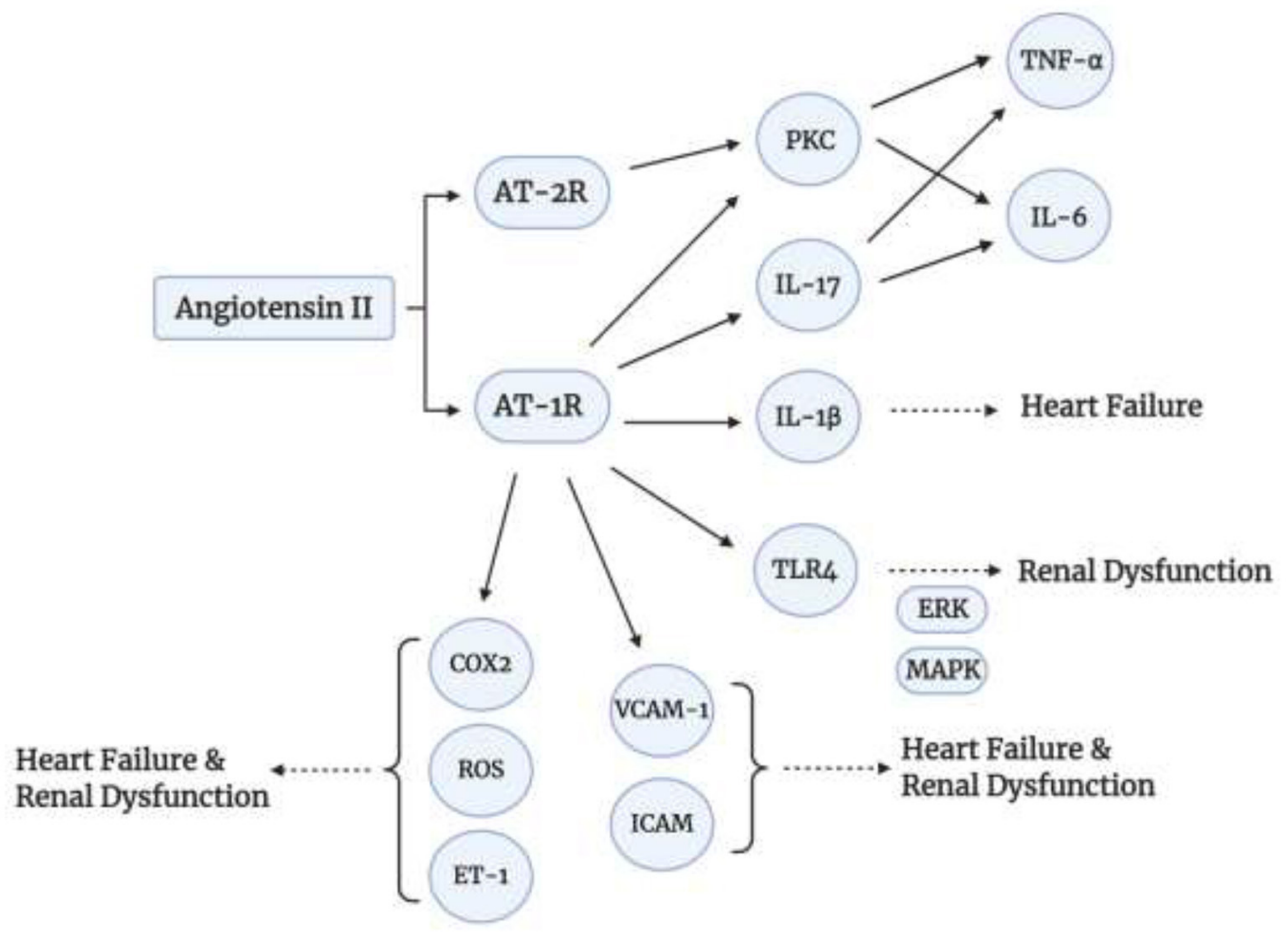
Angiotensin II signaling pathways. Angiotensin II induces inflammatory cytokine production via binding to AT-1 and AT-2.

To add to that, Angiotensin II upregulates the expression of Selectins (P-, E-, and L-selectins), as well as, Vascular Cell Adhesion Molecules-1 (VCAM-1) and Intracellular Adhesion Molecule-1 (ICAM-1), via TNF-α (55, 60). These markers are elevated in Chronic Kidney Disease and Chronic Heart Failure (60). Angiotensin II also increases the kidney expression of Endothelin 1 (ET-1) (61), which stimulates vasoconstriction, inflammation, and fibrosis (cardiac remodeling) (62). Thus, this expression is increased in hypertension, heart failure, and kidney disease. Another role of Angiotensin II in oxidative stress, apoptosis, and inflammation is via Toll-Like Receptor 4 (TLR-4) expression via binding to AT-1 receptor (55). The TLR4 signaling inflammatory cascade mediates renal dysfunction via phosphorylation of Extracellular Receptor Kinase (ERK) and Mitogen Activated Protein Kinase (MAPK) (63). The MAPK signaling is also activated by Angiotensin II. Furthermore, Angiotensin II plays a role in oxidative stress via Cyclooxygenase 2 (COX-2) activation to generate vasoactive prostaglandins and ROS which will play a role in endothelial dysfunction (55). It also generates ROS upon binding to its AT-1 receptor via NADPH oxidase (NOX) (55). A summary of some signaling pathways of Angiotensin II is shown in Figure 3.

### JAK/STAT Pathway

IL-6, which is also activated by Angiotensin II, is secreted as a result of ischemia, and binds to plasma membrane receptors which leads to a signal transduction pathway activating JAK and STAT proteins (64). This signaling pathway has an important role in diabetic nephropathy via an Angiotensin II- dependent mechanism, and is negatively regulated by Suppressor of Cytokine Signaling 3 (SOCS3) by either inhibiting the JAK tyrosine kinase activity or by competing with STATs on cytokine receptors (65). This means SOCS3 negatively regulates IL-6. Another protein mediated by STAT3 is Ephrin-B2, which is increased in diabetes (66). Ephrin-B2 stimulates cardiac fibrosis by the activation and interaction of STAT3 and TGF-β/SMAD3 signaling pathways (67). IL-6 and TNF-α are also regulated by the interaction between Lipopolysaccharide (LPS) and Cluster of Differentiation 14 (CD14) via activation of Nuclear Factor Kappa B (NF-κB) signaling (68).

## ROS Induced Inflammatory and Cardiorenal Syndromes in Diabetes

**Figure 4:**
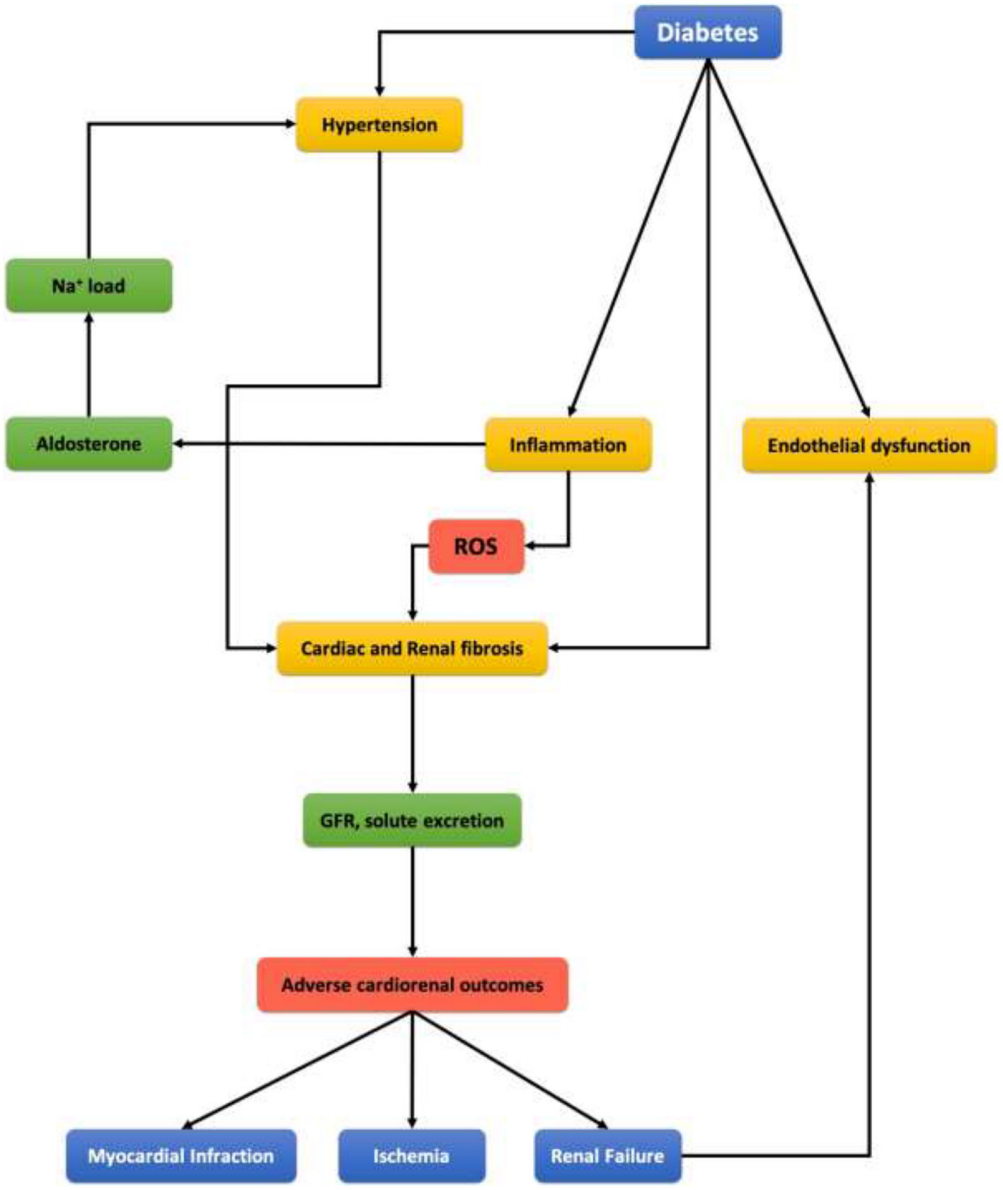
Representing scheme of clinical link between diabetes mellitus, cardiovascular disease, and chronic kidney disease.

Oxidative Stress is described as an imbalance between oxidants (like ROS) and antioxidants (like NO), which results in an accumulation of the oxidants (69). So, it is when the production of oxidants (ROS) is greater than the body's antioxidative metabolic ability. ROS play a major role in hypertension, cardiovascular disease, and renal damage, which emphasizes their contribution to cardiorenal syndrome (as shown in Figure 4) (55). ROS are small molecules derived from oxygen; they are generated in several cellular processes. One major way is Angiotensin II-induced activation of NADPH oxidase, as we mentioned before.

Nicotinamide Adenine Dinucleotide Phosphate (NADPH) oxidase, also known as NOX, plays a major role in ROS formation via utilizing NADPH as an electron donor to reduce oxygen and produce superoxide anions (${\text{O}}_{2}^{-}$) and Hydrogen Peroxide (H_2_O_2_) (70). The high levels of oxygen radicals lead to mitochondrial dysfunction by inactivating mitochondrial enzymes and cause DNA damage at the cellular level (71). At the organ level, our focus will be on the cardiorenal axis and markers implicated. The imbalance between ROS and NO is one of the most reliable markers of oxidative stress. The decrease in NO can be due mainly to two things: Reaction of NO with oxygen radicals and high concentrations of Asymmetric Dimethyl Arginine (ADMA) (33). Superoxide anion (${\text{O}}_{2}^{-}$) reacts with NO to form Peroxynitrite (ONOO^−^), the accumulation of which results in vasoconstriction, inflammation, and impaired vascular and renal functions (70). This decrease in NO inhibits P450 enzymes and leads to the production of vasoconstriction molecules (72) via enhancing Cyclooxygenase activity, which promotes the production of Thromboxane A2 (TxA2) (vasoconstrictor) (72). Also, Peroxynitrite increases Thromboxane synthase activity and thus TxA2, so it increases the vasoconstrictor; and inhibits Prostacyclin synthase and thus decreases Prostacyclin-2 production (vasodilator); which leads to an imbalance between the vasoconstrictors and vasodilators which contributes to the pathogenesis of both the heart and the kidney (72). Another factor contributing to the decrease of NO is the high concentration of circulating ADMA, which is an endogenous inhibitor of Nitric Oxide Synthase (NOS) and is highly observed in renal failure (73).

On the other hand, in heart failure, mitochondrial dysfunction plays a role via Angiotensin II by upregulating NOX2; we also have a Mitochondrial-ROS independent pathway by Angiotensin II that results in the upregulation of NOX4 (71). In addition to that, ROS activates Matrix Metalloproteinase (MMP) in cardiac fibroblasts, which leads to structural changes in the myocardium (74). This leads to cardiac remodeling, decrease in contractility, dysfunctional Calcium handling and eventually heart failure (74). Moreover, Hyperuricemia, which is the accumulation of uric acid mainly due to malfunctioning Xanthine Oxidase (XO), also leads to oxidative stress via a dysfunction in the release of ROS and NO. This is associated with kidney disease, heart disease and diabetes (75). ROS also has proinflammatory effect by releasing cytokines; in addition to profibrotic effect by inducing Epithelial-Mesenchymal Transition (EMT) via the Mitogen-Activated Protein kinase (MAPK) activation or SMAD signaling and this will lead to renal fibrosis, in addition to cellular hypertrophy via Extracellular Receptor Kinase (ERK1/ERK2) pathways (70). These most important proinflammatory cytokines are TL-6 and TNF-alpha, and the main transcription factor responsible for initiating the proinflammatory response is NF-kB, the expression of which is increased in diabetic experimental models (76). So, the downstream effects of NF-kB are proinflammatory via MCP-1, TNF-alpha, and IL-6 regulation. Furthermore, at the level of the mitochondria, excess-glucose leads to increased glucose-derived pyruvate oxidation which increases the number of electron donors in the electron transport chain resulting in over production of superoxide (76).

## Antioxidants and Anti-Inflammatory Approaches to Treat Diabetes-Induced Cardiorenal Syndrome

As discussed previously and evidenced in the literature, oxidative stress has a huge role in injury and pathogenesis of cardiorenal syndrome (77). Therefore, it logically entails that antioxidants have a possible protective ability that would prevent cardiorenal syndrome. The role of oxidative stress and antioxidants appears clearly in diabetes mellitus related cardiorenal syndrome (type 5) (78). Therefore, we will focus in this part on evidence that has explained and proven the protective role of antioxidants in diabetes mellitus and type 5 cardiorenal syndrome. Since the 1960s, the role of antioxidants in improving health and well-being was discovered by biologists (79). In the next decade, Cameron and Pauling (80) were able to find that ascorbic acid (vitamin C) is a potential human cancer protective agent. Since then, antioxidants have become a hot topic in medical research and scientists are looking deeper into the mechanisms, molecular targets, and molecular interactions of antioxidants (81). The antioxidant defense mechanisms are divided into 2 categories: enzymatic and non-enzymatic strategies. Enzymatic antioxidants include superoxide dismutase, catalase, glutathione peroxidase, and glutathione reductase. While non-enzymatic antioxidants include the vitamins A, C, and E, glutathione, α-lipoic acid, mixed carotenoids, coenzyme Q10 (CoQ10), several bioflavonoids, antioxidant minerals (copper, zinc, manganese and selenium), and cofactors like folic acid, uric acid, albumin (82).

In his experiment, Kunisaki et al. (83) was able to show that when he administered vitamin E to diabetic rats, the retinal blood flow and PKC activity in the vascular tissue were normalized. Also, another two short-term experimental studies proved that high doses of vitamin C and lipoic acid can improve some aspects of endothelial dysfunction in diabetes (84, 85). Furthermore, it has been recently reported that vitamin E has the ability to reduce the oxidative stress that builds up in the macrophages in diabetic mice (86). Finally, other studies were able to find prophylactic effects of vitamin E on heart failure patients that have type 1 diabetes. They were able to show that the “supplementation of streptozotocin-induced (STZ)-diabetic rats with 2000 IU of vitamin E/kg of feed beginning immediately after induction of DM and continuing for 8 weeks provided significant protection against cardiac dysfunction induced by T1DM” (87).

As previously revisited mesenchymal stem cells “MSCs”; these cells have the capacity to self-renew rendering them an important immunomodulatory approach to autoimmune diseases (88). MSCs produce soluble factors that can increase the production of anti–inflammatory cytokine interleukin (IL)−10 and decreased production of interferon–gamma (IFN–γ) and IL−12 through altering the secretion of dendritic cells (DCs) (89, 90) MSCs engage the inhibitory molecule programmed death 1 (PD−1) to its ligands PD–L1 and PD–L2 thus, suppressing T–cell proliferation (90, 91). This control over T-cells including CD4^+^CD25^+^FoxP3^+^ (90), has influence on the susceptibility to diabetes induction (92) Moreover MSCs, through the production of soluble factors can also inhibit the proliferation and secretion of B cells (90). This release of trophic and immunomodulatory factors by MSCs seems to hold the therapeutic capacity of these cells (90).

Another therapeutic approach for the cardiorenal syndrome is via the Chinese herbal medication Qiliqiangxin (QLQX). QLQX is composed of 11 different herbs that are alismatis rhizome, carthami flos, cinnamomi ramulus, ginseng radix et rhizome, astragali radix, citri reticulatae pericarpium, salvia miltiorrhiza radix et rhizome, aconiti lateralis radix preparata, semen descurainiae lepidii, periploca cortex, and polygonati odorati rhizome (93). It has been shown that QLQX can be used for regulating the immune response and improving circulation via the Astragali Radix component and acts by reducing the production of TNF-α (93). In addition to that, QLQX has a similar effect as Olmesartan by inhibiting the AT-1 receptor; and thus, inhibits the Ang II-induced cardiac fibroblasts' transdifferentiation via reducing IL-6 transcription and regulating nuclear activity of Nuclear Factor of Activated T-cells (NFAT3) (94). By that, QLQX attenuates cardiac inflammatory reactions and protects myocardial structure and function in HF (94). On the other hand, QLQX can also protect against renal injury in cardiorenal syndrome (CRS) by regulating the oxidative stress and inflammation signaling (95). QLQX significantly reduced inflammatory cytokines and AT receptors in the kidney reducing the inflammatory response; in addition to, reducing the ROS content and thereby regulating the oxidative stress response (95).

Additionally, a study has shown that QLQX improves autophagy via TRPV-1 dependent mechanism in the diabetic heart (9). It also showed that QLQX treatment improves cardiac function and myocardial phenotype in the diabetic mouse model (96). To add to that, QLQX improves endothelial aortic function in diabetic rats via RAS and NO pathways; by inhibiting the expression of ACE and AT-1, and by regulating the NO balance (97).

## Conclusion

CRS defines the different clinical conditions in which heart dysfunction and kidney dysfunction overlap, it describes the negative effects of an impaired renal function on the heart and circulation. In this review, we have thoroughly explained CRS beginning from epidemiological data and classifications aiming to clarify the historical lack of a clear definition due to the complexity of this disease. Immunomodulation encompasses all therapeutic interventions aimed at modifying the immune response as such offering innovative drug targets and methods for immunomodulatory interventions. A better understanding of CRS holds the promise of regenerative medicine, which points toward repairing damaged tissues, stimulating the healing mechanism of organs and implanting laboratory grown tissues when the body is unable to heal itself.

## Author Contributions

AE and FH conceived and designed the review article and approved the final version to be submitted. LA, MN, and NS performed the literature review and wrote the first draft of the review. HG discussed, assembled the data, and revised the manuscript for intellectual content. NA worked on the figures of this review. All authors contributed to the article and approved the submitted version.

## Conflict of Interest

The authors declare that the research was conducted in the absence of any commercial or financial relationships that could be construed as a potential conflict of interest.

## Abbreviations

CRS: Cardiorenal Syndrome
RAS: Renin Angiotensin System
JAK: Janus kinase
STAT: Signal Transducer And Activator of Transcription
DM: Diabetes Mellitus
MSC: Mesenchymal stem cells
DCM: Diabetic Cardiomyopathy
DN: Diabetic Nephropathy
mAb: Monoclonal antibody
ACT: Adoptive cellular therapy
HFpEF: Heart failure with preserved ejection fraction
FFAs: Free fatty acids
LGI: Low-grade inflammation
TNF-α: Tumor necrosis factor-alpha
NF- κB: Nuclear Factor Kappa B
TGF-β: Transforming Growth Factor-Beta
IFN-γ: Interferon-gamma
IL: Interleukin
Treg: T-regulatory cells
Th17: T-helper 17
CAD: Coronary artery disease
GFR: Glomerular filtration rate
GBM: Glomerular basement membrane
LXA4: Lipoxin 4
ADQI: Acute Dialysis Quality Initiative
HF: Heart failure
RBF: Renal blood flow
SNS: Sympathetic nervous system
NADPH: Nicotinamide Adenine Dinucleotide Phosphate
NOX: Nicotinamide Adenine Dinucleotide Phosphate oxidase
NOS: Nitric Oxide Synthase
ACE: Angiotensin Converting Enzyme
AT-1: Angiotensin II type 1 receptor
AT-2: Angiotensin II type 2 receptor
PKC: Protein Kinase C
AP-1: Activator Protein 1
Cx43: Connexin 43
VCAM-1: Vascular Cell Adhesion Molecules-1
ICAM-1: Intracellular Adhesion Molecule-1
ET-1: Endothelin 1
TLR-47: Toll-Like Receptor 4
ERK: Extracellular Receptor Kinase
MAPK: Mitogen Activated Protein Kinase
COX-2: Cyclooxygenase 2
ROS: Reactive Oxygen Species
SOCS3: Cytokine Signaling 3
LPS: Lipopolysaccharide
CD14: Cluster of Differentiation 14
ADMA: Asymmetric Dimethyl Arginine
TxA2: Thromboxane A2
MMP: Matrix Metalloproteinase
XO: Xanthine Oxidase
EMT: Epithelial-Mesenchymal Transition
MAPK: Mitogen-Activated Protein kinase
CoQ10: Coenzyme Q10
STZ: Streptozotocin-induced
DCs: Dendritic cells
PD-1: Programmed death 1
QLQX: Qiliqiangxin.
